# The association between obesity and quality of life: a retrospective analysis of a large-scale population-based cohort study

**DOI:** 10.1186/s12889-021-12009-8

**Published:** 2021-11-03

**Authors:** J. Stephenson, C. M. Smith, B. Kearns, A. Haywood, P. Bissell

**Affiliations:** 1 School of Human & Health Sciences, University of Huddersfield, Queensgate, Huddersfield, HD1 3DH UK; 2 School of Health and Related Research, University of Sheffield, Sheffield, UK

**Keywords:** Obesity, Quality of life, Long-term conditions

## Abstract

**Background:**

The relationship between obesity and health-related quality of life (HRQoL) may be confounded by factors such as multimorbidity. The aim of the study was to explore this relationship, controlling for long-term conditions and other health, lifestyle and demographic factors in a general adult population. There was specific interest in the impact of high weight status, measured by body mass index (BMI) levels (obesity, morbid obesity) compared with individuals of normal weight.

**Methods:**

Health, lifestyle and demographic data were collected from 64,631 individuals aged 16 years and over registered in the Yorkshire Health Study; a long-term cohort study. Data were collected in 2 waves: from patients attending GP surgeries in the South Yorkshire region; and using online recruitment across the entire Yorkshire and Humber area. Univariable and multivariable regression methods were utilised to identify factors associated with HRQoL as measured by the EQ-5D summary score. Long-term conditions were tested as both covariates and mediating factors on the causal pathway between obesity and HRQoL.

**Results:**

Increasing levels of obesity are associated with reduced HRQoL, although this difference is negligible between those of normal weight and those who are overweight. Individuals with obesity and morbid obesity score 4.9 and 11.3 percentage points less on the EQ-5D summary scale respectively than those of normal weight. Concurrent physical, and particularly mental health-related long-term conditions are substantively related to HRQoL: those with 3 or more reported mental or physical health conditions score 29.8 and 14.6 percentage points less on the EQ-5D summary scale respectively than those with fewer conditions. Long-term conditions can be conceptualised as lying on the causal path between obesity and HRQoL, but there is weak evidence for a partial mediating relationship only.

**Conclusions:**

To conclude, in agreement with the established literature we have found a clear inverse relationship between increasing weight status and decreasing HRQoL and confirmed the mediating role of long-term conditions in the reduction of HRQoL in people with obesity. Nevertheless, a high BMI remains independently related to HRQoL, suggesting that ‘healthy people with obesity’ may be in transition to an unhealthy future.

## Background

Health-related quality of life (HRQoL) is a broad subjective concept that encompasses both physical and mental health, which are themselves in complex relationships with other external factors such as health, socio-economic status, the environment and other factors [1]. Obesity is a condition of ‘abnormal or excessive fat accumulation that may impair health’, defined by the WHO [2] as a body mass index (BMI) greater than 30 kg/m^2^, with a BMI of more than 40 kg/m^2^ defined as morbid obesity. The aetiology of obesity is complex and multifaceted, stemming from biological, behavioural and environmental causes [3].

Worldwide obesity has tripled since 1975, and in 2016, 1.9 billion adults (39% of the worldwide adult population) were considered to be overweight: i.e. have a BMI in the range 25 kg/m^2^ ≤ BMI < 30 kg/m^2^; and 650 million (13% of the worldwide population) were considered to have obesity: i.e. have a BMI in the range BMI ≤ 30 kg/m^2^ [2]. In England in 2018, 63% of adults were classified as being overweight or having obesity, with 2 and 4% of men and women respectively being defined as having morbid obesity: i.e. have a BMI in the range BMI ≤ 40 kg/m^2^ [4]. It has been predicted that by 2050 Britain could be a mainly obese society [3]. Connelly reported a noticeable increase in the proportion of the United Kingdom population at very high risk of chronic disease due to their weight [4]. Physical associations include long-term health conditions such as Type 2 diabetes, hypertension, dyslipidaemia, coronary artery disease, stroke, various cancers, reduced reproductive function, osteoarthritis, liver and gall bladder disease, chronic pain and adverse respiratory effects [3, 5, 6]. The proportion of individuals reporting long-term conditions (LTCs) has been shown to increase linearly with increasing BMI, and to be independently related to BMI, after adjusting for age and gender [7]. Similarly, the number of reported LTCs increases with BMI, with 25 and 42% of individuals with moderate and morbid obesity respectively reporting 3 or more LTCs, compared with 12% of normal weight individuals. In addition to physical disease, obesity is also associated with mental health conditions: sleep disorders, anxiety, depression low self-esteem, motivational disorders, eating disorders, impaired body image [1, 8–10] and serious psychiatric disorders [10, 11].

Obesity is associated with physical, mental and economic consequences. The economic consequences of obesity are substantial and increasing [12]. In the UK alone it is estimated that by 2050 the societal and business costs of obesity will reach £49.9billion per year [3]. These costs have been categorised by Seidall [13] as direct costs from treating obesity and its related diseases; societal costs arising from loss of work due to increased absence, physical limitations, lower life expectancy and unemployment benefits; and personal costs stemming e.g. from stigmatisation and discrimination leading to lower incomes and higher healthcare costs. Physical and mental long-term conditions can impact both on each other and Health Related Quality of Life [6, 14–16], and the relationship between obesity and HRQoL can be both mediated and confounded by the presence of comorbidities [17, 18] and other effects such as medication [11] and polypharmacy [19].

The Yorkshire Health Study (YHS) is an observational cohort study of health and lifestyle in Yorkshire and the Humber [20, 21] supported by NIHR CLAHRC (Collaboration for Leadership in Applied Health Research and Care). Adults (aged 16 and over) residing in the in the Yorkshire and Humber region of England are eligible to enter.

The data, from 70,836 adults, was collected in two waves: the first 27,813 were recruited via GP surgeries in South Yorkshire between 2010 and 2012; the second wave of data collection, from 2013 to 2015 utilised online recruitment and the National Clinical Research Network to recruit 43,023 participants. The majority of participants, whether recruited in Waves 1 or 2, completed one survey only. It is well established that there is an inverse relationship between QoL and obesity [12, 17, 22–24]. There are many research studies that demonstrate improved quality of life following both dietary and surgical weight loss [25–27].

## Methods

The aim of this study was to utilise a large, contemporary cohort from the UK to explore the relationships between obesity and HRQoL, controlling for LTCs and other health, lifestyle and demographic factors in a general adult population; considering specifically the impact of high levels of BMI (obesity and morbid obesity) in comparison to BMI levels corresponding to individuals of normal weight.

Personal (age, gender, academic history, employment status, socio-economic status, quality of life), health (history of diabetes, physical and mental long-term conditions, frequency of visits to health care professionals, frequency of visits to hospital, days off work due to sickness) and lifestyle (smoking status, weekly levels of walking and exercise) data were collected from participants who responded to either Wave 1 and/or the full version of the questionnaire administered in Wave 2 of the YHS.

HRQoL, as measured by the EQ-5D summary index (measured on a scale from 0 to 1, with higher values representing higher QoL, and derived from scores on individual EQ-5D domains of mobility, self-care, activities, pain and anxiety), was considered to be the outcome measure in the current investigation. The key predictor variable was weight status, measured using BMI, categorised for the purposes of the current investigation as *Normal weight* (18 kg/m^2^ ≤ BMI < 25 kg/m^2^); *Overweight* (25 kg/m^2^ ≤ BMI < 30 kg/m^2^), *Obese* (30 kg/m^2^ ≤ BMI < 40 kg/m^2^), and *Morbidly obese* (BMI ≥ 40 kg/m^2^). This variable was collected in both waves of the survey. Individuals with BMI less than 18 kg/m^2^ were not included in the analysis, as BMIs in this range may be indicative of illness or eating disorder. An investigation into the relationship between QoL and BMI using the first wave only of the YHS [17] revealed the relationship to be monotonic and approximately linear in individuals with BMI values of 18 kg/m^2^ or more: inclusion of underweight individuals’ results in a curvilinear effect.

Additionally, a number of variables, also collected in one or both waves of the survey, were collected and examined for potential inclusion as covariates in the analysis (Table 1). The first mentioned category of the categorical variables above was considered to be the reference category in all cases.

**Table 1:** Potential controlling variables included in the analysis

Variable	Categories/unit
Gender	Male, female
Highest academic qualifications	Below degree level and Degree level or above
Family history of diabetes	No family history of diabetes and Family history of diabetes
Employment status	Not employed and Employed
Presence of long-term conditions (LTCs) associated with both mental health (ie. tiredness/fatigue, insomnia, anxiety/nervousness, depression, memory problems) or physical health (diabetes, breathing problems, hypertension, heart disease, osteoarthritis, stroke, cancer)	Less than 3 mental health/physical health conditions reported and 3 or more mental health/physical health conditions reported
Smoking status	Never smoked/ ex-smoker, and Daily/occasional smoker
Age	Years
Index of multiple deprivation (IMD) score	Score
Time spent in physical exercise (including, for example, swimming, cycling, gym workout, playing football) in week preceding data collection	Hours
Time spent walking in week preceding data collection	Hours
Number of visits to a healthcare professional (including, for example, GP, nurse, physiotherapist, psychologist, social worker etc.) in previous 3 months	Count
Time off work due to sickness in previous 3 months	Days
Visits to hospital as outpatient in previous 3 months	Count
Visits to hospital as day case in previous 3 months	Count
Nights spent in hospital as inpatient in previous 3 months	Count

In addition to modelling the LTC variables as covariates in a multiple regression model, these variables were assessed for their effect as mediating variables on the causal pathway between BMI and QoL; in the light of findings by Doll et al. [7] that the proportion of individuals reporting LTCs, and the number of reported LTCs are significantly predicted by BMI in controlled models.

Physical exercise (including activities such as swimming, playing football, cycling and aerobics) and walking time (including walking to work, to shops and leisure walking) in the week preceding data collection were estimated using the mid-point of options presented as ranges of times (none; 0–1 h per week; 2–3 h per week etc.) offered to respondents as response categories.

The data set was checked before analysis for errors. Any values outside of theoretical or plausible ranges were deleted or replaced with a limiting value as appropriate, with limits for inclusion of BMI values obtained using guidelines. The extent and nature of data missingness was investigated. Missing values were assessed for nature of missingness using Little’s test for data missing completely at random (MCAR) and separate variance *t*-tests and cross-tabulations. Data missing at random (MAR) was inferred if the MCAR test was statistically significant but missingness could be predicted from variables other than the outcome variable from separate variance *t*-tests and cross-tabulations. Following verification of missing data on key variables to be MCAR or MAR, complete case analysis was used with respect to both the key predictor variable (weight status as measured by BMI category) and the outcome measure (EQ-5D score) with no imputation conducted on these variables. Controlling variables with more than 5% missing values on remaining cases were dropped from further analysis. Controlling variables with less than 5% missing values that could be shown or inferred to be MCAR or MAR were imputed using expectation maximisation.

The data were summarised descriptively, by weight status (BMI category) and as a full cohort. A series of simple (univariable) regression models were conducted on valid cases, with imputation where necessary and appropriate, considering both the key variable of weight status and each controlling variable in turn as predictors. Controlling variables showing some substantive relationship with the outcome measure were carried forward for inclusion in a subsequent main effects multiple linear regression analysis alongside weight status. Included variables were assessed for collinearity and regression assumptions for the final multiple model were checked post-estimation using residual plots.

Model transferability was assessed by cross-validation. A regression equation was constructed based on a random 80% of cases with model coefficients used to obtain predicted values on the remaining validation sample. The correlation between predicted and actual values in the validation sample was then compared with the corresponding statistic for the main sample; with low or no reductions representing good model transferability.

Ethical approval for the YHS was granted by the NHS Research Ethics Committee (09/H1306/97).

## Results

Valid data were collected on 64,631 individuals. Data checking revealed a small proportion of certain variables with implausible or impossible data values. These were investigated on an individual basis and deleted or amended where necessary.

Calculated BMI values of the cleaned data set ranged from 8.32 to 85.9 kg/m^2^; with a mean value of 26.7 kg/m^2^ (SD 5.50 kg/m^2^). The BMI ranges and corresponding frequencies associated with each original and merged category are summarised in Table 2.

**Table 2:** BMI categorisation frequencies

Weight status	BMI range	Frequency (valid %)^**1**^
Normal weight	18 kg/m^2^ ≤ BMI < 25 kg/m^2^	27,488 (43.1%)
Overweight	25 kg/m^2^ ≤ BMI < 30 kg/m^2^	21,994 (34.5%)
Obese	30 kg/m^2^ ≤ BMI < 40 kg/m^2^	12,676 (19.9%)
Morbidly obese	BMI ≥ 40 kg/m^2^	1678 (2.6%)

^1^Frequencies do not add to 100% due to rounding

A summary of participant characteristics (by weight status) before imputation and variable deletion is summarised in Table 3; with data based on respondents from whom a valid weight status could be deduced.

**Table 3:** summary of participant characteristics (by BMI status)

Variable	Frequency (valid %)				
	Normal weight (*n* = 27,488)	Overweight (*n* = 21,994)	Obese (*n* = 12,676)	Morbidly obese (*n* = 1678)	All valid BMI categories (*n* = 64,631)
Gender (*n* = 64,481)					
Male	8749 (31.9%)	9838 (44.8%)	4628 (36.6%)	399 (23.9%)	23,813 (36.9%)
Female	18,686 (68.1%)	12,107 (55.2%)	8009 (63.4%)	1270 (76.1%)	40,668 (63.1%)
Academic qualifications (*n* = 64,631)					
Degree level or above	3335 (12.1%)	1898 (8.6%)	693 (5.5%)	59 (3.5%)	6038 (9.3%)
Below degree level	24,153 (87.9%)	20,096 (91.4%)	11,983 (94.5%)	1619 (96.5%)	58,593 (90.7%)
Family history of diabetes (*n* = 34,317)					
No diabetes in family	10,622 (72.9%)	7593 (68.9%)	4130 (57.3%)	512 (48.4%)	23,159 (67.5%)
Diabetes in family	3948 (27.1%)	3426 (31.1%)	3081 (42.7%)	546 (51.6%)	11,158 (32.5%)
Employment status (*n* = 48,136)					
Not employed	5029 (24.2%)	4933 (29.6%)	2520 (27.8%)	282 (25.0%)	12,934 (26.9%)
Employed	15,770 (75.8%)	11,724 (70.4%)	6545 (72.2%)	848 (75.0%)	35,202 (73.1%)
Mental health-related LTCs (*n* = 62,539)					
Less than 3 LTCs	25,640 (93.3%)	20,111 (91.4%)	10,680 (84.3%)	1267 (75.5%)	63,917 (90.2%)
3 or more LTCs	1848 (6.7%)	1883 (8.6%)	1996 (15.7%)	411 (24.5%)	6919 (9.8%)
Physical health-related LTCs (*n* = 62,539)					
Less than 3 LTCs	26,942 (98.0%)	21,075 (95.8%)	11,585 (91.4%)	1440 (85.8%)	67,751 (95.6%)
3 or more LTCs	546 (2.0%)	919 (4.2%)	1091 (8.6%)	238 (14.2%)	3085 (4.4%)
Smoking status (*n* = 64,631)					
Never smoked / ex-smoker	23,531 (85.6%)	19,210 (87.3%)	10,700 (86.9%)	1433 (87.6%)	54,208 (86.1%)
Smoke daily/occasionally	3957 (14.4%)	2784 (12.7%)	1620 (13.2%)	203 (12.4%)	8736 (13.9%)
**Variable**		**Mean (SD)**			
Age (years) (*n* = 63,711)	47.3 (18.6)	53.5 (16.7)	52.5 (16.0)	49.6 (14.9)	50.4 (17.7)
IMD score (*n* = 48,119)	20.5 (16.4)	22.2 (16.8)	25.7 (17.8)	29.6 (18.9)	22.3 (17.1)
Hours spent in physical exercise/cycling in typical week (*n* = 34,532)	3.66 (1.65)	3.33 (1.54)	0.852 (1.32)	0.570 (1.10)	1.25 (1.56)
Hours spent walking in typical week (*n* = 60,029)	2.89 (0.976)	2.75 (1.02)	1.60 (1.08)	1.46 (1.10)	1.73 (1.05)
Visits to healthcare professional in last 3 months (*n* = 64,631)	2.32 (4.92)	2.58 (5.56)	3.26 (5.83)	4.91 (9.19)	2.68 (5.53)
Days off work due to sickness in last 3 months (*n* = 41,446)	2.62 (12.2)	3.36 (14.4)	5.38 (18.4)	10.1 (25.3)	3.59 (14.8)
Visits to hospital as outpatient in last 3 months (*n* = 64,631)	0.567 (2.09)	0.672 (2.10)	0.831 (2.43)	1.12 (3.56)	0.142 (1.15)
Visits to hospital as day case in last 3 months (*n* = 64,631)	0.116 (1.07)	0.150 (1.19)	0.177 (1.24)	0.204 (1.39)	0.814 (2.62)
Nights spent in hospital as inpatient in last 3 months (*n* = 64,631)	0.222 (2.18)	0.222 (1.83)	0.277 (2.63)	0.641 (4.28)	0.247 (2.26)
EQ-5D QoL summary index (*n* = 61,708)	0.848 (0.200)	0.805 (0.224)	0.733 (0.265)	0.619 (0.324)	0.804 (0.232)
EQ-5D QoL score – mobility domain	1.22 (0.572)	1.36 (0.693)	1.58 (0.840)	1.92 (1.04)	1.36 (0.708)
EQ-5D QoL score – self-care domain	1.08 (0.354)	1.11 (0.420)	1.20 (0.556)	1.38 (0.748)	1.12 (0.443)
EQ-5D QoL score – activities domain	1.26 (0.612)	1.35 (0.700)	1.55 (0.849)	1.85 (1.04)	1.36 (0.722)
EQ-5D QoL score – pain domain	1.53 (0.752)	1.70 (0.817)	1.97 (0.941)	2.30 (1.07)	1.70 (0.846)
EQ-5D QoL score – anxiety domain	1.43 (0.710)	1.44 (0.722)	1.61 (0.847)	1.93 (1.02)	1.49 (0.761)

While most differences across groups were statistically significant at the 5% significance level, reflecting the large sample size, few substantive differences across groups were observed. Uni-variable tests of significance revealed low effect sizes (measured by the ϕ and partial-η^2^ statistics) of less than 5% for most reported variables in the table above. However, some cross-group differences of non-negligible magnitude were observed with respect to gender, diabetes status and academic qualifications. A higher proportion of women than men were in the group with morbid obesity; however, overall mean male BMI (26.9 kg/m^2^; SD 4.83 kg/m^2^) was higher than the mean female BMI (26.6 kg/m^2^; SD 5.84 kg/m^2^). The proportion of those in the *Normal weight* group who were qualified to degree level or above was, at 12.1%, more than double that of those in the group with obesity (5.5%) and over 3 times that of those in the group with morbid obesity (3.5%). The proportion of those in the *Normal weight* group who suffered from 3 or more long-term mental health-related conditions was, at 6.7%, less than half that of those in the group with obesity (15.7%) and less than a third that of those in the group with morbid obesity (24.5%).

Little’s test for MCAR using all quantitative variables with complete or near-complete cases revealed no evidence that missing EQ-5D scores were not MCAR (*p* = 0.408). Separate variance t-tests revealed no evidence that missing weight statuses were not MAR. The variables corresponding to diabetes status, employment status, IMD, exercise levels, alcohol consumption and days off work due to sickness were not carried forward for consideration due to excessive proportions of missing values on these variables.

*P*-values, parameter estimates, associated confidence intervals, and effect sizes (using the partial-η^2^ statistic) from a series of univariable regression analyses conducted the outcome measure of EQ-5D score on an imputed data set including the key predictor variable and all controlling variables with complete or near-complete set of cases as identified in Table 3 above, are summarised in Table 4.

**Table 4:** univariable regression model parameters

Variable	***p***-value	Parameter estimate (B)	95%CI for B	Partial-η^**2**^
Weight status (as measured using BMI)				
Normal weight (reference)				
Overweight	< 0.001	− 0.042	(− 0.046, − 0.038)	0.007
Obese	< 0.001	− 0.115	(− 0.120, − 0.110)	0.034
Morbidly obese	< 0.001	− 0.229	(− 0.240, − 0.217)	0.024
Gender				
Male (reference)				
Female	0.018	0.005	(0.001, 0.008)	< 0.001
Age	< 0.001	−0.003	(− 0.004, − 0.003)	0.068
Highest level qualification				
Below degree level (reference)				
Degree level or above	< 0.001	0.100	(0.094, 0.106)	0.016
Mental health-related long-term conditions				
Less than 3 conditions reported (reference)				
3 or more conditions reported	< 0.001	−0.384	(− 0.390, − 0.379)	0.236
Physical health-related long-term conditions				
Less than 3 conditions reported (reference)				
3 or more conditions reported	< 0.001	−0.340	(− 0.349, − 0.332)	0.086
Level of contact with health professions in 3 months	< 0.001	− 0.014	(− 0.014, 0.013)	0.092
Smoking status				
Non-smoker or ex-smoker (reference)				
Current smoker	< 0.001	−0.052	(−0.058, − 0.047)	0.006
Number of hours per week spent walking	< 0.001	0.061	(0.059, 0.062)	0.071
Number of day case hospital visits in last 3 months	< 0.001	−0.019	(−0.018, − 0.021)	0.009
Number of outpatient hospital visits in last 3 months	< 0.001	− 0.022	(− 0.023, − 0.022)	0.046
Number of nights spent as hospital inpatient in last 3 months	< 0.001	−0.012	(− 0.013, − 0.011)	0.014

A mediation analysis revealed that both of the variables modelling mental or physical health-related LTCs exhibited some mediating effect on the relationship between weight status and HRQoL. All paths in the mediation models considering weight status as a predictor, and the mental or physical health-related LTCs in turn as mediators were significant. Path coefficients for weight status were revealed to be − 0.010 in a univariable regression of QoL on weight status; − 0.007 in a model including the variable modelling mental health LTCs and − 0.007 in a model including the variable modelling physical health LTCs. Hence while conditions for partial mediation were met, the conditions were full mediation were not met. The substantive mediating effect was low and weight status continued to significantly predict the outcome in the presence of the mediating variable. Hence analysis proceeded with LTCs being modelled as a controlling covariate.

The simple regression models suggested that age, presence/absence of long-term conditions, level of contact with health professions in last 3 months, number of hours per week spent walking, and number of hospital outpatient visits in previous 3 months should be included alongside a weight status category in a multiple model. As strong evidence for statistical significance was expected in most cases due to the size of the data set, assessments for inclusion were made primarily on the basis of effect sizes, with an associated partial-η^2^ statistic of about 0.025 or more considered to indicate grounds for inclusion of a particular variable. As the predictor variable of key contextual interest, this did not apply to any of the weight status categories. Model parameters from this multiple model are summarised in Table 5.

**Table 5:** multiple regression model parameters

Variable	***p***-value	Parameter estimate (B)	95%CI for B	Partial-η^**2**^
Weight status				
Normal weight (reference)				
`Overweight	< 0.001	−0.013	(− 0.016, − 0.009)	0.001
Obese	< 0.001	− 0.049	(− 0.053, − 0.045)	0.010
Morbidly obese	< 0.001	−0.113	(− 0.123, − 0.104)	0.009
Age	< 0.001	− 0.002	(− 0.002, − 0.002)	0.029
Mental health-related long-term conditions				
Less than 3 conditions reported (reference)				
3 or more conditions reported	< 0.001	−0.298	(−0.303, − 0.293)	0.177
Physical health-related long-term conditions				
Less than 3 conditions reported (reference)				
3 or more conditions reported	< 0.001	−0.146	(−0.154, − 0.138)	0.023
Level of contact with health professions in 3 months	< 0.001	− 0.007	(− 0.008, − 0.007)	0.040
Number of hours per week spent walking	< 0.001	0.033	(0.032, 0.035)	0.032
Number of outpatient hospital visits in last 3 months	< 0.001	−0.009	(−0.009, − 0.008)	0.011

The R^2^ and adjusted-R^2^ statistics for this model were both 0.390; representing a moderately good fit to the data. No evidence for collinearity was revealed, with variance inflation factors all within tolerable limits. Analysis of residuals revealed no clear evidence for violations of regression assumptions, with normally distributed standardised residuals which exhibited no clear pattern when plotted against standardised predicted values. The model showed very good cross-validation properties, with negligible loss in correlation computed from the validation sample fitted values against predictions from the training sample model coefficients.

Hence controlling for other categorical factors and covariates, compared to individuals in the *Normal weight* category; HRQoL was essentially the same in individuals in the *Overweight* category; slightly lower (4.9 percentage points less on the EQ-5D summary index) in individuals in the *Obese* category and lower (11.3 percentage points less on the EQ-5D summary index) in individuals in the *Morbidly obese* category. Hence the effect of morbid obesity, compared to normal weight, has approximately the same impact as 3 or more physical long-term conditions or an increase in age of about 55 years. Amongst the controlling variables, those with the greatest substantive effect on QoL were mental and physical health-related LTCs: those with 3 or more mental health conditions scored 29.8 percentage points less on the EQ-5D summary index than those with 2 or fewer conditions; and those with 3 or more physical health conditions scored 14.6 percentage points less on the EQ-5D summary index than those with 2 or fewer conditions. Higher quality of life was also reported by younger people, by those who saw health professionals more infrequently and spent less time visiting hospital as an outpatient, and by those who spent more time walking.

## Discussion

### Key findings

The analysis has revealed a clear relationship indicating lower levels of QoL with weight status defined by categories of increasing BMI in individuals with BMIs in the range of 18 kg/m^2^ and above. This monotonic decrease in QoL, recorded in groups categorised by increasing BMI, is consistent with both the findings relating to the individual EQ-5D items in the analysis by Kearns et al. [17] of the first wave of the YHS data, and the wider literature [12, 23]. The effect on QoL of weight status category is substantial, particularly for those in the highest BMI category. This reduction in QoL as a result of increasing BMI is greater than that found linked to cancer, myocardial infarction and diabetes, and similar to having schizophrenia, heart failure or kidney failure (Sullivan 2001). However, the EQ-5D summary index is a highly negatively skewed measure, with about one third of our respondents scoring the maximum value of 1.00 and over half of respondents scoring 0.84 or more.

Comparing the estimates and magnitudes thereof of the weight status variables in the simple and multiple models reveals that the effect of weight status is smaller in the multiple (controlled) model. The variables corresponding to mental and physical health-related LTCs in the multiple model appear to be of greater effect on QoL than weight status itself. This may be due to a proportion of the residual variance ascribed to weight status in the simple model being ascribed to other variables in the multiple model; specifically, LTCs, which are already known to be related to weight status from the descriptive analysis and is reflected in the 2007 Sach analysis of BMI and quality of life. It may also reflect the status of obesity as a risk factor for many LTCs [3, 5–8]. However, there are no changes of direction of association of parameter coefficients or substantial changes in parameter estimates or inferences of significance between the models. Further work considering the impact of specific individual conditions may be beneficial.

The mediation analysis reveals that the presence of mental or physical health-related LTCs has a limited partial mediating effect on the underlying relationship between weight status and QoL. In the current analysis, LTCs are analysed as controlling factors. Nonetheless, LTCs can alternatively be conceptualised as lying on the causal path between BMI and QoL [1, 10, 17]; although the direct link between BMI and QoL is stronger and more intuitive. Further model-testing work is needed to establish the existence of, and direction of associations between other constructs represented in the YHS.

The unique contribution of BMI to QoL is consistent with Scottish data [18] which found an independent relationship between obesity and Quality of Life. This is in contrast to the ‘Healthy Obesity’ hypothesis and may represent a subset of the population ‘in transition’ to unhealthy obesity [28] via metabolic syndrome, not measured in our study.

The largest unique effect in the multiple model was the presence of 3 or more mental health LTCs. This may be an artefact of the data, explained by a presumed higher likelihood of MH LTCs being related in our sample, compared to the ‘independence’ of the physical domains of LTC. The second biggest effect is degree of contact with a health professional, which we presume is acting as a proxy measure for general health.

### Strengths and limitations

The strengths of the YHS are its large sample size which allows for an exploration of detailed obesity categories, comprehensive examination of a wide range of variables, and the use of EQ-5D which measures HRQoL using public preferences.

Most measures captured by the YHS are self-reported and may not be completely reliable; particularly those requiring accurate recall, such as activity levels or levels of contact with healthcare professionals over an extended period of time; or the ability of respondents to distinguish between, for example, hospital visits as an out-patient or day case. The key predictor of BMI requires accurate self-reporting of both height and body weight in appropriate units. In addition, self-reported height and weight are respectively over and underestimated in both men and women (Niedhammer 2000, Spencer 2002, Taylor 2006). In the current study, analysis was restricted to variables which were derived from items elicited in both waves of the questionnaire.

The fit of the multiple regression model to the data, though of moderately high magnitude, may have been constrained in magnitude by uncertainties in the integrity of certain measures and the limited availability of variables for which an acceptable proportion of valid cases were available. Nonetheless, a moderately good fit was obtained and cross-validation procedures revealed that model portability is good; it should be expected that the model will perform equally well on samples other than that from which parameter coefficients were derived.

### Implications for future work

This study has demonstrated that further work is needed to establish the existence of, and direction of associations; for example, it seems plausible that not only can factors such as BMI and exercise impact on quality of life (as was assumed in this analysis), but also that variables such as exercise level and BMI are correlated with a plausible association in either direction. A number of models are required to be tested for model fit using, for example, a confirmatory factor analysis approach in order to ensure that an optimal series of relationships are tested.

## Conclusions

To conclude, in agreement with the established literature we have found a clear inverse relationship between increasing weight status and decreasing QoL, using a large regional cohort study. We have investigated the influence of other demographic, lifestyle and health related domains on this relationship and confirmed the mediating role of LTCs in the reduction of QoL in people with obesity. Nevertheless, a high weight status remains independently related to QoL, suggesting that the ‘healthy obese’ may be in transition to an unhealthy future.

## Data Availability

Anonymised data and details regarding using the resource for recruiting participants to studies can be gathered by contacting Professor Elizabeth Goyder (e.goyder@sheffield.ac.uk). Multi-disciplinary collaboration is strongly encouraged.

## Abbreviations

BMI: Body mass index
HRQoL: Health-related quality of life
QoL: Quality of life
LTC: Long-term conditions
